# Collectanea Medica: Consisting of Anecdotes, Facts, Extracts, Illustrations, &c.

**Published:** 1823-11

**Authors:** 


### COLLECTANEA MEDICA:

CONSISTING OF

### ANECDOTES, FACTS, EXTRACTS, ILLUSTRATIONS, &c.

*Relating to the History or the Art of Medicine, and the Collateral Sciences.*

Floriferis, ut apes, in saltibus omnia libant,

Omnia nos, itidem, depascimur aurea dicta.

ART. I.—*Extracts from "Medical Jurisprudence."* By J. A. PARIS, M.D. &c. and J. S. M. FONBLANQUE, Esq.

### *Of Impositions.*

UNDER this head we shall comprehend the subject of Feigned Diseases and that of the Adulterations of Food.

*Feigned, or Simulated Diseases.*—There are several objects, for the accomplishment of which persons are induced to simulate the existence of disease: such as, for obtaining military exemptions and discharges, or certain civil disqualifications; for the purpose of deriving parochial relief, or pecuniary assistance from benefit societies, or the comfortable shelter and retreat of an hospital; for exciting compassion and obtaining alms; for creating public interest and curiosity; for procuring release from confinement, or exemption from punishment; and, lastly, for the dishonest intention of recovering unjust compensation from some person selected for accusation, as the author of the pretended calamity.

The subject has been very ably treated by different authors on Medical Jurisprudence, especially by Mahon and Foderé, whose opportunities for observation during the severe operation of the conscription-laws must have been numerous and instructive: in our own country, the work of Dr. Hennen, on the Principles of Military Surgery, will be found to contain some very valuable information upon the detection of such impostures.

The diseases which have been selected for the accomplishment of any of the purposes above enumerated are extremely numerous, although there are some few which may be said to be more generally preferred on such occasions.

In general, the medical inquirer will not have much difficulty in detecting such impositions; although there are cases where the investigation becomes a subject of extreme delicacy and importance, as in those of persons reporting themselves sick and unfit for military service, or *malingerers*, as they are technically called. It must be confessed that there is a degree of eclat attending the detection of a fraud, which is very likely to lead the practitioner astray, by inducing him to attach undue importance to the supposed proofs of guilt: such cases have unfortunately occurred, and the innocence of the party has been compromised by the vanity of the inquisitor.

Whenever the suspicions of a medical person are excited with respect to the sincerity of a patient's account, he should always endeavour to conceal them; he should become himself a dissembler, "superare malitiam malitia;" for, while the impostor is persuaded that the medical attendant is his dupe, he will be less on his guard. He should then be desired to describe with minuteness every symptom and circumstance of his malady; he should be questioned as to its origin, progress, and duration, its seat and intensity, and the effects produced upon it by remedies. Few imposters will be able to withstand such interrogatories without tripping; they will soon betray some incongruity in their statements, and enable the pathologist to elicit the truth. A girl of seventeen counterfeited epilepsy so well in the general hospital of Montpellier, as to elude all suspicion, until M. de Sauvages, who, being less credulous, asked her whether she had not felt an air pass from the hand to the shoulder, and from the shoulder to the thigh? when, upon her replying in the affirmative, he ordered her to be whipped; after which she had never any return of the disease. If a patient complains of a long protracted disease, which has rendered his life uncomfortable, and we at the same time perceive that his body has not undergone emaciation, we are naturally led to suspect the truth of his statement; and we shall find little difficulty in verifying or dispelling our suspicions: nor ought we to forget, in an inquiry of this nature, to learn whether the patient has in truth ever flown to any remedy for relief; for, if he be an impostor, however cheerfully he may have appeared to submit to medical discipline, we shall find, upon minute examination, that he has uniformly neglected every plan proposed for his cure. Galen was, from a circumstance of this kind, led to the detection of a person who feigned a fit of cholic, in order to avoid attending a public assembly; but he was observed to neglect the remedy *(philonium,)* which had uniformly relieved him when labouring under the actual attack of the disease, to which he was in reality subject. We should, moreover, be informed respecting the previous character, habits, constitution, and former complaints, of the suspected invalid; and we should learn the ostensible reasons which the individual in question may have for feigning ill health, whether for temporary or permanent purposes. The inspections should be conducted in private; for it has been remarked, by those most experienced in these subjects, that the number of spectators always increases the obstinacy of the impostor.

When the more ordinary modes of investigation have failed in leading to the detection of an imposture, of whose existence we entertain but little doubt, we may proceed to a system of intimidation, and to severe discipline. Few impostors, however sturdy, can withstand the cravings of hunger, blistering, the affusions of cold water, and, above all, a continual nausea from the administration of divided doses of tartarized antimony; and yet exceptions of an extraordinary kind might be adduced. "I have seen an instance (says Dr. Hennen,) where the patient admitted of all the preparatory measures of amputation before he thought proper to relax his knee-joint." The same author also relates the case of a dragoon who bore very severe riding-school duty for some weeks, secured to his horse, before he could be brought to acknowledge that his chronic rheumatism was assumed. Mahon records a very extraordinary instance of a conscript, who feigned blindness, and baffled every attempt to detect the imposition: he was even placed on the margin of a river, and desired to go forward, which he did, and fell into the stream. He was, however, without doubt, aware that boats were provided for his safety; for, after having received his discharge, he freely acknowledged the imposition which he had practised.

Having offered these general remarks, we shall proceed to consider the particular diseases more usually counterfeited, and the modes best calculated for their detection; although we must here observe that, after all that can be said upon the subject, each case will require an exertion of ingenuity for its detection, for which no previous instruction can ever provide.

*Insanity* has in all ages been feigned for the accomplishment of particular objects: we read of its having been thus simulated by David, Ulysses, and Lucius Brutus. * * In general, the detection of such an imposture will not be difficult: the feigned maniac never willingly looks his examiner in the face, and, if his eye can be fixed, the changes in his countenance, on being accused, will be strongly indicative of his real state of mind. It is, moreover, very difficult to imitate the habits of a lunatic for any length of time, and to forego sleep. An insane person generally sleeps but little, and talks much during the night; but the pretender, if he thinks he is not watched, will sleep, and only act his part when he believes his conduct to be observed.

*Somnolency.*—This is a state of body which the sturdy impostor has in several instances assumed: he pretends to be in a state incapable of any muscular motion; he is constantly in bed, retaining that posture in which his limbs are placed, or may happen to fall; his great aim is to appear unconscious of the external world. The interesting case of this kind related by Dr. Hennen must be considered as the master-piece of imposture. A person of the name of Drake, in the Royal African Corps, assumed an appearance of total insensibility, under which he resisted every kind of treatment: he resisted the shower-bath, as well as shocks of electricity; but, on a proposal being uttered in his presence to apply the actual cautery, his pulse rose; and, on preparations being made to remove him to Bethlem hospital, an amendment soon manifested itself.

*Syncope.*—It seems probable that certain persons have possessed a controlling power over the action of the heart. Dr. Cleghorn, of Glasgow, mentions in his lectures the case of a person, whom he knew, who could feign death, and had so completely the power of suspending, or at least moderating, the action of the heart, that its pulsation could not be felt: this man, it appears, some years afterwards, died suddenly. The story of Colonel Townshend is well known, who, in the presence of Dr. Cheyne and some other physicians, put on all the appearance of death, and was resuscitated of his own accord. In this instance it is said that neither pulse nor respiration could be perceived for more than half an hour: he, however, actually died on the same evening.

Dr. Hennen relates a most interesting case of violent palpitation of the heart, which was produced by the man's own efforts. Dr. Hennen found that he could at any time render the affection very imperfect by throwing the patient's head well back, so as to destroy that voluntary combination of muscular action, which he believes to have produced the palpitation. "We must suppose (says he,) that this person had the power of throwing the muscles which narrow the chest into sudden and strong action, at the moment when the apex of the heart made its stroke upwards." After a serious admonition, Dr. Hennen sent the man back to his duty; and, as he afterwards remained without any murmur or complaint, we must consider his obedience as a tacit acknowledgment of his guilt. Some persons have pretended that they have no pulsation at the wrist, and they occasion its cessation by prussure on the artery, or by taking a full inspiration, and continuing to retain the breath as long as possible.

*Epilepsy.*—There is, perhaps, no disease that has been more frequently simulated with success: its characters, and mode of attack, offer great facilities for the impostor; it does not require the unremitting caution which other maladies exact for successful imitation; nor is it necessary, as Dr. Smith observes, to assume it but at convenient times, it being perfectly consistent with the nature of the disorder to be quite well in the intervals, which may be longer or shorter at the impostor's pleasure. During the feigned attack, the blood is generally sucked from the gums, and the mouth made to froth by chewing soap. There is, however, one symptom of the disease which cannot be imitated,—the incontractility of the pupil of the eye on exposure to light, which in a real fit of epilepsy is always dilated and immoveable: nor is the patient affected by rubbing stimulants on the nose. During these feigned convulsions, impostors have often suffered the most flagrant liberties to be taken with their persons, without betraying the least consciousness of what was going on; such as having pins and needles run into different parts of their bodies. This fact admits, in some degree, of physiological explanation. Compression on the muscles, by acting on their nervous filaments, or by some unknown influence on the distribution of nervous energy, renders them less sensible in proportion as they become contracted: wounds are thus often inflicted in the field of battle which are scarcely felt during a desperate conflict, on account of the high muscular energy of the part which is in force at the time. Indeed, it may be satisfactorily shown that convulsions, or inordinate muscular contractions, are in themselves instinctive efforts to diminish pain.

*Hysteria.*—On account of the variety and mutability of the symptoms which characterise this affection, but little skill is required for its simulation. Dr. Cullen is said to have been deceived by a man, who, pretending to be affected with this disease, was retained in the Edinburgh infirmary as long as suited his convenience, and afterwards triumphantly acknowledged the deceit. Affusion of cold water, low diet, and blisters, will generally furnish the means of detection.

The *shaking Palsy* is a frequent plea on the part of an idle beggar, and is always suspicious, especially where the person appears to be in other respects in an ordinary state of vigour. This ingenious order of mendicants, however, says Dr. Gordon Smith, understands the art of mimicking wretchedness too well not to have the details of their appearance in some degree of keeping.

Before we quit the subject of spasmodic diseases, it is essential to remark that, owing to circumstances and peculiarities of temperament, these diseases assume, on certain occasions, and in particular individuals, an extravagance of character which might create a suspicion of their being feigned. Lord Monboddo, in his "Ancient Metaphysics," mentions an extraordinary case of what he calls "jumping ague," in which the person affected would jump on chairs and tables, and run with great velocity during sleep. Sir John Sinclair, in his statistical account of Scotland, relates also many well-authenticated histories of the same disease; and in some parts of Forfarshire it is said to be extremely common; and there is reason to believe that it may be propagated by a species of sympathy. Numerous are the instances on record where the accidental sight of a patient suffering an epileptic attack, has immediately occasioned a similar attack on the spectator; so that epilepsy has been supposed to be sometimes communicable from one person to another, nearly in the same manner as has been observed of the action of yawning, and agreeably to a notion alluded to by the poet,

"Dum spectant oculi læsos, læduntur et ipsi."

Similar spasmodic diseases have been occasioned by religious enthusiasm, and, propagated by sympathy, have become in a very wonderful manner epidemic. In such cases, although we must consider those in whom the affection originated as designing impostors, we are bound to acquit the general mass of sufferers of any blame, except that which may attach to excessive credulity.

*Fever.*—The state of the system after a night's debauch may deceive a person unaccustomed to such inspections. Emetics have also been taken with the same view, and the face has been exposed to the fumes of sulphur. Foderé likewise states that paleness has been induced by smoking cummin-seeds; and we have heard that a paroxysm of fever may be excited and kept up by the introduction of a clove of garlic into the rectum. Dr. Hennen says that he has seen many attempts to simulate fever by whitening the tongue with chalk, &c.; and he has often met with old soldiers profoundly versed in the history of a paroxysm of intermittent, and very skilful in imitating the rigors. The detection, however, of such artifices cannot be difficult.

*Dropsy.*—This is more generally feigned by pregnant women. * * Sauvages relates the case of a mendicant who gave to his child the appearance of hydrocephalus, by piercing the integuments of the head, and gradually introducing air; and Ambrose Paré mentions a similar practice for the purpose of counterfeiting hydrocele.

*Jaundice.*—If any attempt should be made to colour the skin yellow, the whiteness of the tunica conjunctiva, as well as the appearance of the urine and fæces of the patient, will always detect the imposition.

*Hæmopthysis.*—This disease has been frequently feigned by sucking blood from the cheeks, gums, &c. but the professional inspector can never be deceived by such artifices: the appearance of the sputa, the state of pulse, &c. will always indicate the truth. Besides which, detection must be insured by a careful examination of the mouth and fauces.

[To be continued.]

